# RETRACTION: “Influence of Rosiglitazone on the Expression of PPAR*γ*, NF‐*κ*B,and TNF‐*α* in Rat Model of Ulcerative Colitis”

**DOI:** 10.1155/grp/9859572

**Published:** 2026-06-08

**Authors:** Gastroenterology Research and Practice

**RETRACTION**: J‐W. Mao, H‐Y. Tang, and Y‐D. Wang, “Influence of Rosiglitazone on the Expression of PPAR*γ*, NF‐*κ*B,and TNF‐*α* in Rat Model of Ulcerative Colitis,” *Gastroenterology Research and Practice*, no. 2012 (2012). 10.1155/2012/845672.

The above article, published online on 17 October 2012 in Wiley Online Library (http://wileyonlinelibrary.com), has been retracted following the agreement of the journal’s Associate Editor, Haruhiko Sugimura; and John Wiley & Sons Ltd.

The retraction has been agreed to following an investigation of concerns initially raised by Elizabeth Bik on PubPeer [1], which identified multiple instances of inappropriately overlapping figures. Our investigation concluded that there are concerns about the reliability of this article as follows:

‐ Figure 3: The images of the Control group after 7 days and the Rosiglitazone group after 14 days appear highly similar to the model and Etiasa‐treated groups after 14 days shown in Figure 4 of [2], a previous article by the same author group.

‐ Figure 4: The images of the mucosal tissue in the Model group after 14 and 35 days share unexpected similarities. In addition, the image of the tissue in the Control group after 35 days appears identical to the Etiasa‐treated tissue after 56 days shown in Figure 4 of [2].

In light of the above, the findings of the article can no longer be considered reliable. Therefore, the article is retracted.

The authors disagree with this retraction.

## References

[1] Bik E. M. , Influence of Rosiglitazone on the Expression of PPAR*γ*, NF-*κ*B,and TNF-*α* in Rat Model of Ulcerative Colitis, PubPeer. September 2024, https://pubpeer.com/publications/1699B4ACE296657C446A5E2853313D.

[2] Mao J.-W. , Tang H.-Y. , Tan X.-Y. , and Wang Y.-D. , [Retracted] Effect of Etiasa on the Expression of Matrix Metalloproteinase-2 and Tumor Necrosis Factor-*α* in a Rat Model of Ulcerative Colitis, Molecular Medicine Reports. (2025) 31, no. 6, 148, 10.3892/mmr.2025.13513. 40183370 PMC11980537

